# Preclinical multimodality phantom design for quality assurance of tumor size measurement

**DOI:** 10.1186/1756-6649-11-1

**Published:** 2011-09-30

**Authors:** Yongsook C Lee, Gary D Fullerton, Cristel Baiu, Margaret G Lescrenier, Beth A Goins

**Affiliations:** 1 Department of Radiology, University of Texas Health Science Center at San Antonio, San Antonio, TX, USA; 2 Department of Radiology, University of Colorado Denver, Aurora, CO, USA; 3 Gammex, Inc., Middleton, WI, USA

## Abstract

**Background:**

Evaluation of changes in tumor size from images acquired by ultrasound (US), computed tomography (CT) or magnetic resonance imaging (MRI) is a common measure of cancer chemotherapy efficacy. Tumor size measurement based on either the World Health Organization (WHO) criteria or the Response Evaluation Criteria in Solid Tumors (RECIST) is the only imaging biomarker for anti-cancer drug testing presently approved by the United States Food and Drug Administration (FDA). The aim of this paper was to design and test a quality assurance phantom with the capability of monitoring tumor size changes with multiple preclinical imaging scanners (US, CT and MRI) in order to facilitate preclinical anti-cancer drug testing.

**Methods:**

Three phantoms (Gammex/UTHSCSA Mark 1, Gammex/UTHSCSA Mark 2 and UTHSCSA multimodality tumor measurement phantom) containing tumor-simulating test objects were designed and constructed. All three phantoms were scanned in US, CT and MRI devices. The size of test objects in the phantoms was measured from the US, CT and MRI images. RECIST, WHO and volume analyses were performed.

**Results:**

The smaller phantom size, simplified design and better test object CT contrast of the UTHSCSA multimodality tumor measurement phantom allowed scanning of the phantom in preclinical US, CT and MRI scanners compared with only limited preclinical scanning capability of Mark 1 and Mark 2 phantoms. For all imaging modalities, RECIST and WHO errors were reduced for UTHSCSA multimodality tumor measurement phantom (≤1.69 ± 0.33%) compared with both Mark 1 (≤ -7.56 ± 6.52%) and Mark 2 (≤ 5.66 ± 1.41%) phantoms. For the UTHSCSA multimodality tumor measurement phantom, measured tumor volumes were highly correlated with NIST traceable design volumes for US (R^2 ^= 1.000, p < 0.0001), CT (R^2 ^= 0.9999, p < 0.0001) and MRI (R^2 ^= 0.9998, p < 0.0001).

**Conclusions:**

The UTHSCSA multimodality tumor measurement phantom described in this study can potentially be a useful quality assurance tool for verifying radiologic assessment of tumor size change during preclinical anti-cancer therapy testing with multiple imaging modalities.

## Background

Highly consistent, reproducible and standardized response criteria are essential to evaluate the efficacy of new anti-cancer drugs in multicenter trials [1]. The World Health Organization (WHO) criteria and the Response Evaluation Criteria in Solid Tumors (RECIST) have been widely used as the only imaging biomarker presently approved by the United States Food and Drug Administration (FDA) for drug testing, although the use of functional imaging methods such as Positron Emission Tomography (PET) Response Criteria in Solid Tumors (PERCIST) complements the limitations of anatomic methods in treatment response assessment in terms of biological relevance and prognostic information [2-4]. The criteria require either two dimensional (WHO criteria- sum of the product of greatest perpendicular dimensions in the transverse plane over all target lesions) or one dimensional (RECIST - sum of single longest dimensions in the transverse plane for arbitrary five lesions per organ and up to ten lesions per patient) tumor size measurements [1,4-12]. Three-dimensional radiologic assessment of tumor burden also has been performed using volumetric techniques [13,14].

Several quality assurance (QA) phantoms for anatomic measurement have been developed for assessment with computed tomography (CT) and magnetic resonance imaging (MRI) [15-22]. However, the development of these phantoms has predominately focused on clinical assessment. Yet, in recent years, there has been an emphasis to improve preclinical anti-cancer drug testing by incorporating longitudinal imaging of tumor models with use of preclinical scanners specially designed for small rodents [23-25]. A tumor measurement QA phantom for preclinical studies in rodent models could be used to identify and correct biased measurement results for tumor size determined with different imaging modalities in multiple laboratories or institutions [26]. In addition, the verification for radiologic assessment of tumor size change using this QA phantom would allow standardization of imaging protocols prior to animal studies, thus potentially reducing the number of animals required, increasing study efficiency and decreasing cost.

This Technical Advance describes the evolution in design, construction and testing of a multimodality QA phantom for use with preclinical scanners. Initial design attempts modified commercial phantoms available for human testing. By using the results from these early versions, the UTHSCSA multimodality tumor measurement QA phantom was successfully constructed for further quality assurance testing of tumor size in rodent models.

## Methods

### Gammex/UTHSCSA Mark 1 phantom

**Figure 1:**
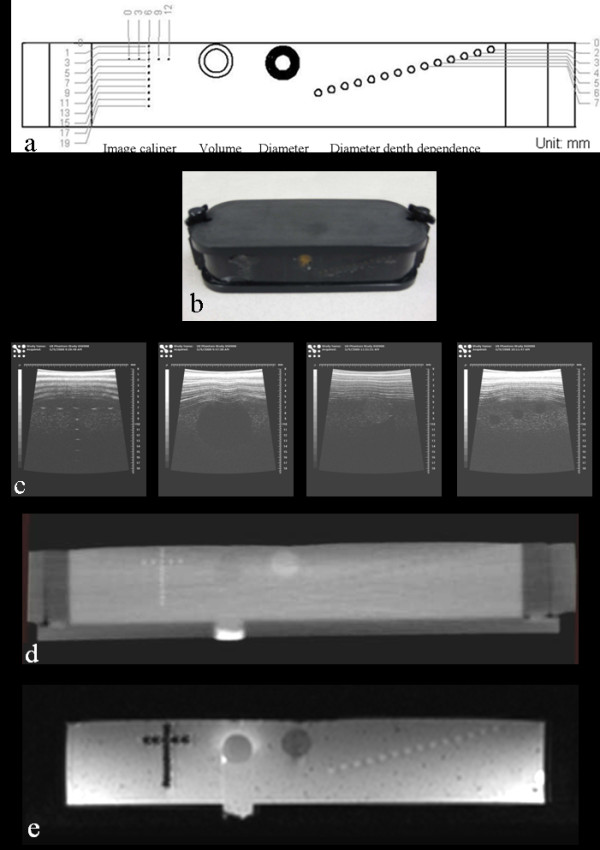
**Gammex/UTHSCSA Mark 1 phantom**. (a) Design, (b) photograph, (c) image caliper, volume, diameter and diameter depth dependence of US images, (d) CT and (e) T1 weighted MR images of the phantom are displayed. Visualsonics US unit with 35 MHz frequency, clinical Philips CT and clinical Philips 3T MRI units were used. The size of the phantom was too large to fit into the bore of all preclinical CT and MR scanners.

In 2007, the Gammex 404 GS LE phantom was modified in an attempt to construct the first generation tumor measurement phantom and this new phantom was denoted as Gammex/UTHSCSA Mark 1 phantom (Figure 1 a and 1b, Table 1). The phantom was composed of four sets of measurement calibration standards: A. Image Caliper - stainless steel wires at 2 mm vertical intervals and 3 mm horizontal intervals, B. Volume - two spheres with volumes 179.59 and 523.59 mm^3^, C. Diameter - long cylinder (5 to 10 mm by 0.5 mm intervals) and D. Diameter Depth Dependence - 2 mm-cylinders from 2 to 15 mm depth.

**Table 1:** Comparison of Gammex/UTHSCSA Mark 1 phantom, Gammex/UTHSCSA Mark 2 phantom and UTHSCSA multimodality tumor measurement phantom

	Gammex/UTHSCSA Mark 1 phantom	Gammex/UTHSCSA Mark 2 phantom	UTHSCSA Multimodality tumor measurement phantom
**Size (cm**^**3**^**)**	16.8 × 7.8 × 4.0	12.0 × 4.3 × 5.0	11.5 × 3.8 × 2.4

**Weight (g)**	537.00	308.49	101.02

**Material**	Gammex TM materials	Gammex TM materials	Water-based TM materials

**Components**			
Image Caliper	Stainless steel wires at 2 mm vertical intervals and 3 mm horizontal intervals	N/A	N/A
Volume	Two low contrast spheres with volumes 179.59 and 523.59 mm^3^	Five low contrast spheres with volumes 4.2 to 1436.8 mm^3^	Five low contrast spheres with volumes 4.2 to 1436.8 mm^3^
Diameter	Low contrast cylinders (5 to 10 mm by 0.5 mm intervals)	Five low contrast spheres (2, 4, 7, 10 and 14 mm)	Five low contrast spheres (2, 4, 7, 10 and 14 mm)
Diameter Depth Dependence	2 mm-cylinders from 2 to 15 mm depth	N/A	N/A

**Problems**			
Size	Too large to fit into bore of preclinical CT and MR scanners.	Too large to fit into bore of some preclinical MR units.	Adequate.
CT contrast	Poor.	Poor due to lack of contrast agent in TM materials.	Adequate.
Artifact in US	Reverberation artifacts due to the surface membrane material chosen for phantom.	Distortion of spheres was evident because the lateral resolution was significant in areas far from focal length.	Slight reverberation artifacts were observed.

US, CT and MR images of the phantom were obtained following the imaging protocols listed in Table 2 (Figure 1 c-e). The size of test objects in the phantom was measured three times independently. For US, visual measurements were made using a measurement tool in Vevo 770 v.2.2.3 software (Visualsonics Inc., Toronto, ON, Canada). For MRI, a full width at half maximum (FWHM) method was used in ImageJ software (Version 1.42q, National Institutes of Health, Bethesda, MD). RECIST and WHO analyses were performed according to their definitions. For volume accuracy, the equation V = π/6·a·b·c where a, b and c are diameters in three perpendicular dimensions was used [27].

**Table 2:** Imaging modalities and protocols used in this study

	Gammex/UTHSCSA Mark 1 phantom	Gammex/UTHSCSA Mark 2 phantom	UTHSCSA Multimodality tumor measurement phantom
**US**	Vevo 770™ -120 high frequency system(Visualsonics Inc. Toronto, ON, Canada)	Vevo 770™ -120 high frequency system(Visualsonics Inc. Toronto, ON, Canada)	Vevo 770™ -120 high frequency system(Visualsonics Inc. Toronto, ON, Canada)
	Scanhead RMV 703, 35 MHz center frequency, 10 mm focal length	Scanhead RMV 703, 35 MHz center frequency, 10 mm focal length	Scanhead RMV 703 35 MHz center frequency, 10 mm focal length
Resolution	Axial: 50 μ, Lateral: 110 μ	Axial: 50 μ, Lateral: 110 μ	Axial: 50 μ, Lateral: 110 μ

**CT**	Brilliance 64-slice scanner (Philips Healthcare, Andover, MA)	Micro-CT (Gamma Medica-Ideas, Inc., Northridge, CA)	Micro-CT (Gamma Medica-Ideas, Inc., Northridge, CA)
	Tube voltage: 120 kV, Tube current: 133 mA, Exposure time: 0.75 s, CTDI_vol : _21.4 mGy	Tube voltage: 75 kV, Tube current: 185 μA, Acquisition time: 60 s	Tube voltage: 75 kV, Tube current: 185 μA, Acquisition time: 60 s
Resolution	0.35 × 0.35 × 1.00 mm^3^	0.17 × 0.17 × 0.17 mm^3^	0.17 × 0.17 × 0.17 mm^3^

**MRI**	Achieva 3.0T scanner (Philips Healthcare, Andover, MA)	Intera 1.5T scanner (Philips Healthcare, Andover, MA)	7.0T scanner (Bruker BioSpin MRI GmbH, Ettlingen, Germany)
	Surface coil Turbo spin echo T1 weighted images TR = 1121 ms, TE = 4.63 ms, echo train factor = 5 and number of average = 10	Head coil 3D FFE T1 weighted images TR = 8.59 ms, TE = 4.19 ms, echo train factor = 0 and number of average = 24	Bruker Volume coil 3D Turbo RARE
Resolution	0.94 × 0.94 × 5.00 mm^3^	0.75 × 0.75 × 2.00 mm^3^	0.13 × 0.20 × 0.23 mm^3^

* FFE - Fast Field Echo

* RARE - Rapid Acquisition with Relaxation Enhancement

### Gammex/UTHSCSA Mark 2 phantom

**Figure 2:**
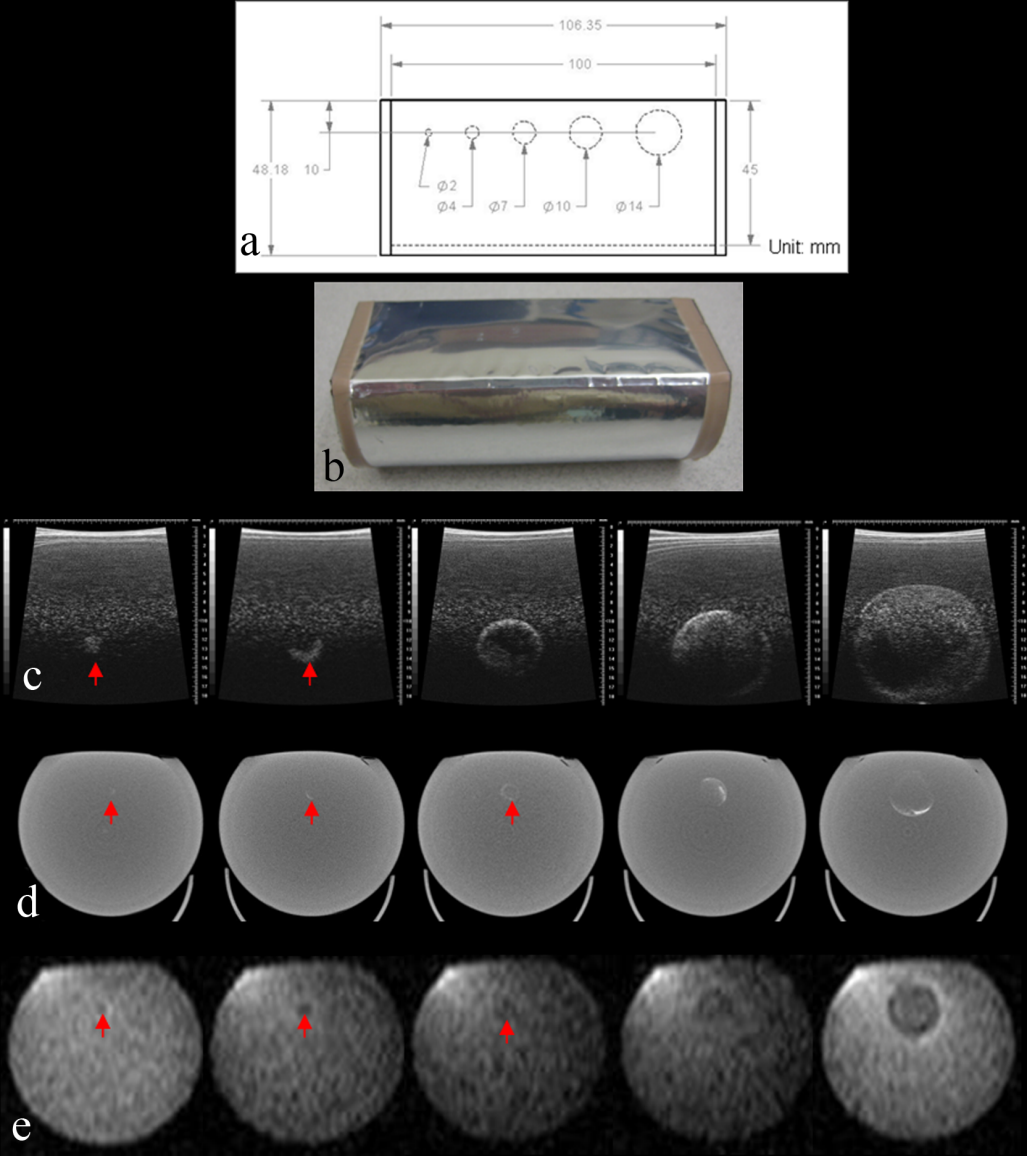
**Gammex/UTHSCSA Mark 2 phantom**. (a) Design, (b) photograph, (c) US, (d) micro-CT and (e) T1 weighted MR images of the phantom are shown. Visualsonics US unit with 35 MHz frequency, Gamma-Medica Ideas micro-CT unit and clinical Philips 1.5 T MRI unit were used. Sphere distortion in US images and poor contrast in CT images show limitations of the phantom (red arrow).

After testing the Gammex/UTHSCSA Mark 1 phantom in multiple imaging modalities, the phantom was redesigned based on the number of target lesions (five per organ) required by RECIST and the dimensions necessary for use in preclinical scanners (Figure 2 a and 2b, Table 1). The phantom consisted of five tumor-simulating test objects with different diameters of measurement calibration standards: A. Diameter - low contrast spheres (2, 4, 7, 10 and 14 mm) and B. Volume - low contrast spheres with volumes 4.2 to 1436.8 mm^3^. The sizes of test objects were chosen based on the following reasons: 2 mm is the smallest tumor size in rodent models that can be readily palpated and 14 mm is the maximum tumor size tolerated without perturbing influence by host animal physiology.

US, CT and MR images were acquired and the size of test objects in the phantom for the US and MR images was measured to calculate RECIST, WHO and volume as described for the Mark 1 phantom (Figure 2 c-e, Table 2).

### UTHSCSA multimodality tumor measurement phantom

**Figure 3:**
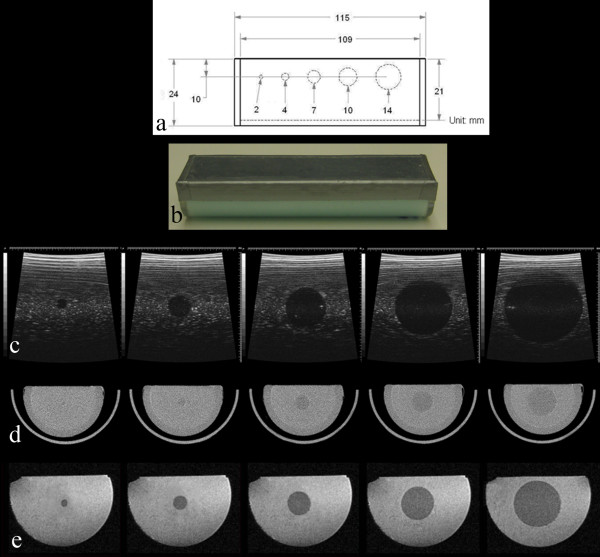
**UTHSCSA multimodality tumor measurement phantom**. (a) Design, (b) photograph, (c) US, (d) micro-CT and (e) T2 weighted MR images of the phantom are shown. Visualsonics US unit with 35 MHz frequency, Gamma-Medica Ideas micro-CT unit and Bruker 7.0 T MRI unit were used. Artifacts, size and contrast problems noted with Gammex/UTHSCSA Mark 1 and Mark 2 phantoms were solved with this phantom.

A new multimodality tumor measurement phantom was constructed to improve the contrast and geometry of Gammex/UTHSCSA Mark 1 and 2 phantoms (Figure 3 a and 3b, Table 1). The phantom had five test objects of 2, 4, 7, 10 and 14 mm as Gammex/UTHSCSA Mark 2 phantom but was constructed with smaller dimensions (length × width × depth of 11.5 cm × 3.8 cm × 2.4 cm) so that it would fit any preclinical scanner. The phantom was made in house of tissue mimicking (TM) materials based on methods developed in Dr. Ernest L. Madsen's laboratory at the University of Wisconsin Madison [28] (See additional file 1: technical appendix with detailed description of phantom construction; additional file 2: Table S1 summarizing the phantom ingredients; additional file 3: Figure S1 describing silicone mold preparation; additional file 4: Figure S2 describing silicone mold procedures; and additional file 5: Figure S3 describing phantom assembly procedures).

US, CT and MR images of the phantom were acquired (Figure 3 c-e, Table 2). The size of test objects in the phantom was measured to calculate RECIST, WHO and volume for the US and MRI images as described for the Mark 1 phantom. For CT, a FWHM method was used in ImageJ software. For CT and MRI, contrast (%) was calculated using the equation C = (S_background _- S_object_)/S_background_.

### Statistical analysis

Linear regression analysis was performed on design volume (NIST traceable gold standard) as a function of measured volume of test objects for Gammex/UTHSCSA Mark 2 phantom and the UTHSCSA multimodality tumor measurement phantom using GraphPad Prism software (Version 5.01, GraphPad Software Inc, San Diego, CA). Analysis of RECIST and WHO for all three phantoms and that of volume for Gammex/UTHSCSA Mark 1 phantom could not be performed because two data points were insufficient for statistical analyses. A p-value < 0.05 was considered statistically significant.

## Results

### Multimodality images of Gammex/UTHSCSA Mark 1 phantom, Gammex/UTHSCSA Mark 2 phantom, and UTHSCSA multimodality tumor measurement phantom

Table 1 summarizes the size, weight, material, components and problems of Gammex/UTHSCSA Mark 1 phantom, Gammex/UTHSCSA Mark 2 phantom, and UTHSCSA multimodality tumor measurement phantom. Figure 1 c - e depicts US, CT and MR images of Gammex/UTHSCSA Mark 1 phantom. During testing of the Mark 1 phantom, several problems became evident. First, although the Mark 1 phantom was initially designed to fit into preclinical scanners, it was too large to fit into the bore of preclinical mouse CT and MR units. Second, CT contrast in the two spheres for volume accuracy needed to be improved for accurate size measurement. Third, reverberation in the surface of the phantom interfered with US imaging.

In the Gammex/UTHSCSA Mark 2 phantom, the design was simplified based on the number (five per organ) of target lesions required by RECIST as shown in Figure 2. The UTHSCSA multimodality tumor measurement phantom had the same structure as the Mark 2 phantom but the geometry and contrast of the phantom were improved by reducing the size of the phantom and adding contrast agents to the TM materials as displayed in Figure 3. Tumor-simulating test objects appeared darker than background in all three images and the contrast between test objects and background (CT: 9.67% and MRI: 25.15%) was sufficient to distinguish test objects and measure their size. Except for a small reverberation close to the surface in the US images, no artifacts were evident for the UTHSCSA multimodality tumor measurement phantom.

### Size measurement in Gammex/UTHSCSA Mark 1, Gammex/UTHSCSA Mark 2 phantom, and UTHSCSA multimodality tumor measurement phantom

RECIST, WHO and volume analyses for two spheres from US and MR images of the Gammex/UTHSCSA Mark 1 phantom are displayed in Table 3. For the Mark 1 phantom, smaller errors were determined for RECIST for both US (1.73 ± 0.44%) and MRI (-2.65 ± 3.74%) compared with WHO (US, -4.75 ± 1.30%; MRI, -7.56 ± 6.52%), with MRI errors larger than for US by both RECIST and WHO. For volume analysis, MRI errors were larger than for US for both the 7 mm and 10 mm test objects. RECIST, WHO and volume analyses for CT were not determined due to inadequate CT contrast.

**Table 3:** RECIST, WHO and volume analyses from US and MR images of Gammex/UTHSCSA Mark 1 phantom

	Design	Image RECIST and WHO (mean ± SD (% error))	
		
		US	MRI
**RECIST (mm)**	17.00	17.29 ± 0.08 (+1.73 ± 0.44%)	16.55 ± 0.64 (-2.65 ± 3.74%)
**WHO (mm**^**2**^**)**	149.00	141.93 ± 1.94 (-4.75 ± 1.30%)	137.73 ± 9.72 (-7.56 ± 6.52%)
			

	**Design**	**Image Volume (mean ± SD (% error))**	
		
		**US**	**MRI**

**Volume (mm**^**3**^**)**	179.59 (7 mm sphere)	180.45 ± 7.51 (+0.48 ± 4.18%)	152.68 ± 30.81 (-14.99 ± 7.16%)
	523.59 (10 mm sphere)	498.54 ± 12.60 (-4.78 ± 2.41%)	485.49 ± 31.68 (-7.28 ± 6.05%)

Mean ± standard deviation (SD) for three repeated measures and % errors in parenthesis are shown for RECIST, WHO and volume analysis. RECIST and WHO calculations were based on their definitions. For volume calculation, an equation V=π/6·a·b·c where a, b and c are diameters in three perpendicular dimensions was used.

Table 4 shows RECIST, WHO and volume analyses for five test objects from US and MR images of the Gammex/UTHSCSA Mark 2 phantom. Measurements from CT images were not determined due to the same reasons as mentioned for the Mark 1 phantom (Figure 2 d). For US, RECIST (5.66 ± 1.41%) had larger errors than WHO (-0.16 ± 1.32%). For MRI, RECIST and WHO analyses showed small errors ranging from 0.39 ± 2.54% for RECIST to -2.05 ± 2.79% for WHO. Volumes calculated from US images had larger errors (range of -5.69 ± 1.59% to 7.29 ± 5.65%) for smaller test objects (2, 4 and 7 mm) which improved with the analyses of the 10 mm (3.99 ± 2.03%) and 14 mm (1.21 ± 0.66%) test objects. Volume analysis from MR images showed similar features to that for US but had much larger errors (range of -21.81 ± 66.60% to 11.86 ± 21.62%) for smaller test objects (2, 4 and 7 mm). For Mark 2 phantom, tumor volume measured by US and MRI correlated (p < 0.0001) with design volume (Table 4). The best fits for US and MRI versus design volume were line y = 1.014 ± 0.009x - 0.152 ± 6.341 (R^2 ^= 0.9998; p < 0.0001) and line y = 0.962 ± 0.011x - 6.665 ± 7.357 (R^2 ^= 0.9996; p < 0.0001), respectively.

**Table 4:** RECIST, WHO and volume analyses from US and MR images of Gammex/UTHSCSA Mark 2 phantom

	Design	Image RECIST and WHO (mean ± SD (% error))	
		
		US	MRI
**RECIST (mm)**	37.00	39.09 ± 0.52 (+5.66 ± 1.41%)	37.15 ± 0.94 (+0.39 ± 2.54%)
**WHO (mm**^**2**^**)**	365.00	355.44 ± 4.69 (-0.16 ± 1.32%)	348.69 ± 9.95 (-2.05 ± 2.79%)
			

	**Design**	**Image Volume (mean ± SD (% error))**	
		
		**US**	**MRI**

**Volume (mm**^**3**^**)**	4.19 (2 mm sphere)	4.37 ± 0.40 (+4.40 ± 9.47%)	3.28 ± 2.79 (-21.81 ± 66.60%)
	33.51 (4 mm sphere)	35.93 ± 1.89 (+7.29 ± 5.65%)	37.46 ± 7.24 (+11.86 ± 21.62%)
	179.59 (7 mm sphere)	169.38 ± 2.85 (-5.69 ± 1.59%)	155.43 ± 29.57 (-13.45 ± 16.46%)
	523.60 (10 mm sphere)	544.50 ± 10.65 (+3.99 ± 2.03%)	484.32 ± 25.94 (-7.50 ± 4.95%)
	1436.76 (14 mm sphere)	1453.39 ± 9.42 (+1.21 ± 0.66%)	1381.45 ± 32.07 (-3.80 ± 2.23%)
	**Slope [95% CI]**	1.014 ± 0.009 [0.984 to 1.043]	0.962 ± 0.011 [0.928 to 0.996]
	**Intercept [95% CI]**	-0.152 ± 6.341 [-20.33 to 20.02]	-6.665 ± 7.357 [-30.08 to 16.75]
	**R**^**2**^	0.9998	0.9996
	**p-value**	< 0.0001	< 0.0001

Mean ± standard deviation (SD) for three repeated measures and % errors in parenthesis are shown for RECIST, WHO and volume analysis. The same analysis methods as described for the Mark 1 phantom were used. Slope and intercepts [95% confidence intervals], correlation coefficient (R^2^) and p-value of linear regression lines for volume of test objects also are shown.

RECIST, WHO and volume analyses for the UTHSCSA multimodality tumor measurement phantom are displayed in Table 5. Unlike results for the Mark 2 phantom, RECIST and WHO calculations showed reduced errors (range of -1.47 ± 0.25% to 1.69 ± 0.33%) for all three modalities. RECIST analysis showed smaller errors than WHO analysis except for CT. For volume analysis, errors were ≤ -2.84 ± 2.49% except for the 10 mm test object in MRI (-5.34 ± 0.76%) and the smallest test object (2 mm) with errors ranging from -18.30 ± 10.65% to 5.72 ± 0.60% for CT and MRI, respectively. For the UTHSCSA multimodality tumor measurement phantom, US-, CT- and MRI-measured tumor volume also correlated (p < 0.0001) with design volume (Table 5). US, CT and MRI -measured volume versus design (NIST traceable gold standard) volume had the best fit of lines y = 0.980 ± 0.003x + 2.277 ± 2.261 (R^2 ^= 1.000; p < 0.0001), y = 1.011 ± 0.004x + 0.413 ± 3.052 (R^2 ^= 0.9999; p < 0.0001) and y = 0.977 ± 0.008x - 1.013 ± 5.613 (R^2 ^= 0.9998; p < 0.0001), respectively. These results demonstrate that technical personnel using the phantom could quickly prove the data from all three modalities is acceptable over the entire range of sizes with error limits determined by the study designer by comparing the slope and intercept values from a simple regression analysis (Table 5).

**Table 5:** RECIST, WHO and volume analyses from US, CT and MR images of UTHSCSA multimodality tumor measurement phantom

	Design	Image RECIST and WHO (mean ± SD (% error))		
		
		US	CT	MRI
**RECIST (mm)**	37.00	37.05 ± 0.05 (+0.13 ± 0.13%)	37.62 ± 0.12 (+1.69 ± 0.33%)	37.39 ± 0.13 (+1.05 ± 0.34%)
**WHO (mm**^**2**^**)**	365.00	359.634 ± 0.93 (-1.47 ± 0.25%)	368.76 ± 1.47 (+1.03 ± 0.39%)	359.96 ± 2.09 (-1.38 ± 0.57%)
				

		**Image Volume (mean ± SD (% error))**		
		
		**US**	**CT**	**MRI**

**Volume (mm**^**3**^**)**	4.19 (2 mm sphere)	3.55 ± 0.00 (-15.34 ± 0.04%)	3.42 ± 0.45 (-18.30 ± 10.65%)	4.43 ± 0.03 (+5.72 ± 0.60%)
	33.51 (4 mm sphere)	32.76 ± 0.33 (-2.24 ± 0.99%)	32.56 ± 0.83 (-2.84 ± 2.49%)	34.05 ± 1.24 (+1.62 ± 3.70%)
	179.59 (7 mm sphere)	180.71 ± 1.71 (+0.62 ± 0.95%)	179.41 ± 4.06 (-0.10 ± 2.26%)	180.84 ± 4.25 (+0.70 ± 2.36%)
	523.60 (10 mm sphere)	520.27 ± 3.19 (-0.64 ± 0.61%)	537.90 ± 4.08 (+2.73 ± 0.78%)	495.65 ± 3.97 (-5.34 ± 0.76%)
	1436.76 (14 mm sphere)	1408.59 ± 5.67 (-1.96 ± 0.39%)	1450.39 ± 11.22 (+0.95 ± 0.78%)	1406.47 ± 8.16 (-2.11 ± 0.57%)
	**Slope [95% CI]**	0.980 ± 0.003 [0.969 to 0.991]	1.011 ± 0.004 [0.997 to 1.025]	0.977 ± 0.008 [0.951 to 1.002]
	**Intercept [95% CI]**	2.277 ± 2.261 [-4.919 to 9.472]	0.413 ± 3.052 [-9.299 to 10.12]	-1.013 ± 5.613 [-18.87 to 16.85]
	**R**^**2**^	1.000	0.9999	0.9998
	**p-value**	<0.0001	<0.0001	<0.0001

Mean ± standard deviation (SD) for three repeated measures and % errors in parenthesis are shown for RECIST, WHO and volume analyses. The same analysis methods as described for the Mark 1 and Mark 2 phantoms were used. Slope and intercepts [95% confidence intervals], correlation coefficient (R^2^) and p-value of linear regression lines for volume of test objects also are shown.

## Discussion

Previous QA phantoms constructed for size measurement had various tumor shapes and focused predominately on measurement of test objects from CT and MRI images using measurement protocols unique to their institution [15-17]. This study focused on construction of a phantom with a simple spherical test object design based on a FDA approved imaging biomarker (WHO criteria, RECIST) for use with multiple preclinical imaging devices. As discussed in Table 1, the Gammex/UTHSCSA Mark 1 and Mark 2 phantoms were too large to fit into the bore of some preclinical CT and MR scanners. Since certain components of the Mark 1 phantom such as image caliper and depth dependence were not required for QA of tumor size measurement, these features were deleted in the Mark 2 phantom based on RECIST (Figure 2). Composite aluminum poly film (Figures 1 c and 2 c) on the Mark 1 phantom surface caused reverberation artifact in the US images that were corrected in future phantoms by using thin composite polyethylene terephthalate/aluminum/linear low density polyethylene (PET/AL/LLDPE). In addition, test objects in US images of the Mark 2 phantom did not appear as perfect spheres compared with those in MR images (Figure 2c). The beam dispersion in the region deeper than focal depth created distortion in the spheres (overestimated diameter in horizontal directions and underestimated diameter in depth). The contrast in CT images of both phantoms was not sufficient to make size measurements (Figure 1 d and 2 d).

In the UTHSCSA multimodality tumor measurement phantom, size, distortion and contrast problems were solved for the images acquired with all three modalities (Figure 3 c-e). First, the diameter of the tumor measurement phantom was reduced to fit within the bore of all preclinical scanners. Second, the center of test objects was designed to be set above the focal depth (10 mm for 35 MHz transducer) to avoid distortion. Third, barium sulfate was used for pronounced CT contrast. As a result, test object measurements were improved for the UTHSCSA multimodality tumor measurement phantom. For all imaging modalities, RECIST and WHO errors were reduced for UTHSCSA multimodality tumor measurement phantom (≤1.69 ± 0.33%) compared with both Mark 1 (≤ -7.56 ± 6.52%) and Mark 2 (≤ 5.66 ± 1.41%) phantoms.

RECIST values were more accurate than WHO values for the UTHSCSA multimodality tumor measurement phantom except for CT. This result corresponded to the fact that WHO criteria are known to give higher risk of measurement error and overestimation of response rates [9]. Volume calculation of the smallest test object (2 mm) in the UTHSCSA multimodality tumor measurement phantom had the largest errors of -15.34 ± 0.04% and -18.30 ± 10.65% for US and CT, respectively, and errors were reduced for larger test objects (≤ -2.84 ± 2.49%) except for 10 mm sphere by MRI (-5.34 ± 0.76%) (Table 5). This explains why small tumors smaller than or equal to 2 mm in preclinical and clinical tumor models cannot be measured with high accuracy.

## Conclusions

The UTHSCSA multimodality tumor measurement phantom design and construction methods provide adequate image quality for validating tumor size measurement in three commonly used preclinical imaging modalities (US, CT and MRI). This tumor measurement phantom provides a potential QA tool for monitoring radiologic assessment of tumor size change in future multi-institutional studies requiring integration of data from disparate sources and devices.

## Competing interests

The authors declare that they have no competing interests.

## Authors' contributions

YCL designed and constructed the UTHSCSA multimodality tumor measurement phantom, acquired images of all phantoms, performed data analysis and drafted the manuscript. GDF conceived the study, designed the phantoms, coordinated phantom construction, data acquisition, and data analysis, and helped to draft the manuscript. CB and MGL designed and constructed Gammex/UTHSCSA Mark 1 and Mark 2 phantoms. BAG participated in the design and construction of UTHSCSA multimodality tumor measurement phantom, and drafted and revised the manuscript. All authors read and approved the final manuscript.

## Pre-publication history

The pre-publication history for this paper can be accessed here:

http://www.biomedcentral.com/1756-6649/11/1/prepub

## Supplementary Material

Additional file 1 **Lee et al Technical Appendix.pdf**. Technical Appendix is a detailed description of the construction of the UTHSCSA multimodality tumor measurement phantom. Click here for file

Additional file 2 **Lee et al Table S1.pdf**. Summary of the ingredients used to construct the UTHSCSA multimodality tumor measurement phantom. Click here for file

Additional file 3 **Lee et al Figure S1.tiff**. Preparation for making silicone molds to cast test objects of UTHSCSA multimodality tumor measurement phantom. (A) Two identical base plates, (B) spacer pairs, (C) steel balls, (D) procedure for gluing steel balls, and (E) two identical mirror image base plates with steel balls. Click here for file

Additional file 4 **Lee et al Figure S2.tiff**. Materials and procedures for making silicone molds. (A) Acrylic rods with 1 mm tips, (B) base plates with fences, alignment rods, acrylic rods, and (C) mold after addition of silicone rubber compound. Click here for file

Additional file 5 **Lee et al Figure S3.tiff**. Procedures for preparing UTHSCSA multimodality tumor measurement phantom using silicone molds. (A) Nylon thread was attached to silicone mold and the molds were adhered with silicone grease. (B) Milk mixture was degassed using house vacuum. (C) After casting test objects in the mold, test objects were mounted in an acrylic container. (D) The top of the container was sealed with surface membrane. (E) Background material was poured into the container. (F) The assembled phantom was placed in rotator and rotated to prevent gravitational sedimentation of tissue mimicking materials. Click here for file
