# Supplementary material for: Preclinical multimodality phantom design for quality assurance of tumor size measurement
Source: BMC Med Phys. 2011 Sep 30;11:1. doi: 10.1186/1756-6649-11-1 (PMC3206432; doi:10.1186/1756-6649-11-1)
Supplement: Additional file 1 — Lee et al Technical Appendix.pdf. Technical Appendix is a detailed description of the construction of the UTHSCSA multimodality tumor measurement phantom. [file 1756-6649-11-1-S1.PDF]

Technical appendix: Construction of UTHSCSA multimodality tumor measurement phantom

A multimodality phantom for tumor size measurement was designed and tested. The half-cylindrical phantom has a length of 11.5 cm and diameter of 3.8 cm to simulate a mouse in preclinical animal scanners. Based on the limited number of target lesions required by Response Evaluation Criteria in Solid Tumors (RECIST), the phantom has five tumor-simulating test objects of different sizes (2, 4, 7, 10 and 14 mm). A test object size of 2 mm was chosen as the smallest size since smaller tumors are not easily palpable and 14 mm was the largest size due to the confounding influence of larger tumors on animal physiology. The following sections provide a detailed description of the phantom construction. First, the design and construction of a mold to prepare the test objects is described. Then instructions are provided for assembly of the phantom.

1. Preparation of silicone molds for casting test objects

1.A. Preparation of base plates for making silicone molds

Silicone molds of test objects were constructed from accurate steel balls glued onto acrylic base plates (Figure S1). Steel balls (14, 10, 7, 4 and 2 mm, accuracy: 2.5 µm) (Nordex Inc., Brookfield, CT) were purchased (Figure S1 C and E). In two acrylic base plates (size: 4.2 cm × 11.5 cm × 0.9 cm, accuracy: 25 µm) (Plastic Supply, San Antonio, TX), five holes (diameter: 14, 10, 7, 4, 2 mm) for test objects and another five holes (diameter: 6 mm) for alignment were made (Figure S1 A and E). Spacer pairs with height of 7, 5, 3.5, 2 and 1 mm (size: 1.0 cm × 5.5 cm, accuracy: 25 µm) were made (Figure S1 B). Two spacer pairs with height of 7 mm and a base plate were placed onto a thin acrylic plate in sequence. Using C-clamps, the

thin plate, spacer pairs and base plate were tightened (Figure S1 D). Steel ball of 14 mm diameter was inserted into 14 mm hole of the base plate and glued using J-B KWIK (J-B WELD Co., Sulphur Springs, TX). The same procedures were performed for the rest of the steel balls. For the other base plate, the steel balls were glued as mirror images (Figure S1 E).

1.B. Preparation of silicone molds

Two silicone molds were prepared using the acrylic base plates containing the steel balls as described in the previous section. Acrylic rods (diameter: 0.8 cm and length: 5.0 cm) with tips (diameter: 1.0 mm and length: 1.0 mm) were prepared to make tiny holes in a silicone mold (Figure S2 A). Fences were made surrounding the base plates using 2.5 cm-high acrylic plates and 3M 2600 masking tape (3M Industrial Adhesives and Tapes, St. Paul, MN). For one base plate, the top of the fence was prepared. The top had five holes (diameter: 0.8 cm) for inserting the acrylic rods and another ten holes (diameter: 1.2 cm) for inserting alignment rods with 1 mm tips and for pouring silicone (Figure S2 B). Steel rods (diameter: 0.9 cm and length: 5.0 cm) were inserted into alignment holes. The acrylic rods were also inserted into the holes and glued using silicone glue to secure the assembly while pouring silicone. Silicone rubber compound (SMOOTH-ON, INC. Easton, PA) was poured into the base plates (Figure S2 C).

2. Construction of UTHSCSA multimodality tumor measurement phantom using silicone molds

2.A. Construction of rotator to rotate phantom

A rotator was constructed to rotate the phantom after construction to ensure a homogeneous tissue mimicking material background (Figure S3 F). A polyvinyl chloride (PVC) pipe was cut to

270 mm to make a rotator. A bolt was attached to the end of the PVC pipe using a nut and a washer to fit a hole for a rotisserie motor. Metal plates were bent to support PVC pipe and to adjust the height of PVC pipe. These metal plates were glued onto a plastic plate using J-B KWIK.

2.B. Preparation and casting of test objects

Test objects of the multimodality tumor measurement phantom were cast using silicone molds. Nylon thread was attached along the center of the half-spheres on the mold without 1 mm holes. Silicone grease (Permatex, Inc, Hartford, CT) was applied to the surface of the two molds (Figure S3 A) and the two molds were put together. Tissue mimicking (TM) material for test objects of the phantom was prepared of ingredients listed in Table S1 [28]. Thimerosal (0.02 g) was dissolved in 10 cc of commercial whole milk. This solution was passed through 20 μm and then 10 μm mesh filters (Small parts Inc., Miramar, FL) to remove any impurities that may have been introduced during processing and packaging. This solution was degassed using house vacuum for 30 seconds at room temperature (Figure S3 B). Next, 0.60 g of dry agarose was dissolved in 10 cc of deionized water (18 MΩ) at room temperature. Tap water should not be used since it may include metal ions which lower the relaxation times. The agarose was used as a gelling agent and T2 modifier. Then 0.79 cc of 1-propanol was added and the mixture was degassed. Propanol increases propagation speed for water to the speed of sound for soft tissues of 1540 m/s. The mixture was heated in a 95 °C water bath (Precision Inc., Winchester, VA, Model: 282, Serial #: 601091552) until the agarose solution cleared. In the meantime, the milk was heated in a 55 °C water bath (VWR Inc., Cornelius, OR, Model: 1212, Serial #: 08119606). The molten agarose solution was then cooled to 55 °C in the same water bath. Then 5 cc of agarose

solution was mixed with 5 cc of milk solution to make a 50-50 volume mixture. The mixture was slowly stirred until homogeneous and any air bubbles were removed from the surface. Then 0.0017 g of ethylene diamine tetraacetic acid (EDTA) and 0.0010 g of copper (II) chloride ($CuCl_2 \cdot 2H_2O$) were added for T1 MR contrast enhancement and the mixture was again stirred sufficiently to ensure homogeneity. The TM material was placed into the molds through 1 mm holes using a 22-gauge needle of a syringe (Becton Dickinson & Co, Franklin Lakes, NJ) (Figure S2 C). The molds were kept in a refrigerator (5º C) for about 30 minutes.

2.C. Preparation of background material and phantom assembly

A multimodality tumor measurement phantom was constructed using test objects cast in silicone molds. A half-cylindrical acrylic container (length: 11.5 cm and diameter: 3.8 cm) was prepared. Two 1 mm holes were made in each side of the container to mount the nylon thread with test objects and one hole of 6 mm was made to pour the background TM material in the container. The test objects with nylon thread were unloaded from the molds and mounted in the half-cylindrical container (Figure S3 C). Thin non-conducting polyethylene terephthalate (PET)/aluminum (AL)/linear low density polyethylene (LLDPE) (thickness: 0.12 mm) was adhered onto the acrylic container using 3M Scotch-Weld DP-100 and 3M duck tape (3M Industrial Adhesives and Tapes, St. Paul, MN), and 1 mm holes were blocked using silicone glue (Figure S3 D). TM material for background was made in a similar manner as for the test objects (Table S1) with the following compositional differences: (1) 2.00 g of dry agarose was added to a room temperature solution of 0.79 cc of 1-propanol and 100 cc of deionized water; (2) 0.103 g of EDTA and 0.06 g of $CuCl_2 \cdot 2H2O$ were added; (3) 1.00 g of barium sulfate ($BaSO_4$) and 0.10 g of glass beads (3000E, Potters Industries, Inc., Parsippany, NJ) were included for CT and US

contrast enhancement, respectively. Background TM material was poured into the 6 mm hole of the container using a small funnel (Figure S3 E). The hole was glued using 3M Scotch-Weld DP-100 and the phantom was rotated at 2 rpm in the rotator (Figure S3 F) for 1 to 2 hours at room temperature to prevent gravitational sedimentation.
