# Supplementary material for: Preclinical multimodality phantom design for quality assurance of tumor size measurement
Source: BMC Med Phys. 2011 Sep 30;11:1. doi: 10.1186/1756-6649-11-1 (PMC3206432; doi:10.1186/1756-6649-11-1)
Supplement: Additional file 2 — Lee et al Table S1.pdf. Summary of the ingredients used to construct the UTHSCSA multimodality tumor measurement phantom. [file 1756-6649-11-1-S2.PDF]

**Table S1 - Ingredients in UTHSCSA multimodality tumor measurement phantom**

| Component | Ingredient | Amount | Product # | Vendor | Location |
|---|---|---|---|---|---|
| **Test Object** | | | | | |
| | Whole Milk | 10 cc | N/A | HEB Grocery | San Antonio, TX |
| | Thimerosal | 0.02 g | T-5125 | Sigma-Aldrich | St. Louis, MO |
| | Agarose | 0.6 g | A-0169 | Sigma-Aldrich | St. Louis, MO |
| | Deionized Water (18 MΩ) | 10 cc | N/A | N/A | N/A |
| | 1-Propanol | 0.79 cc | 33538 | Sigma-Aldrich | St. Louis, MO |
| | EDTA | 0.0017 g | 431788 | Sigma-Aldrich | St. Louis, MO |
| | $CuCl_2$ $2H_2O$ | 0.0010 g | 459097 | Sigma-Aldrich | St. Louis, MO |
| **Background** | | | | | |
| | Whole Milk | 100 cc | N/A | HEB Grocery | San Antonio, TX |
| | Thimerosal | 0.2 g | T-5125 | Sigma-Aldrich | St. Louis, MO |
| | Agarose | 2 g | A-0169 | Sigma-Aldrich | St. Louis, MO |
| | Deionized Water (18 MΩ) | 100 cc | N/A | N/A | N/A |
| | 1-Propanol | 0.79 cc | 33538 | Sigma-Aldrich | St. Louis, MO |
| | EDTA | 0.103 g | 431788 | Sigma-Aldrich | St. Louis, MO |
| | $CuCl_2$ $2H_2O$ | 0.06 g | 459097 | Sigma-Aldrich | St. Louis, MO |
| | $BaSO_4$ | 1 g | B8675 | Sigma-Aldrich | St. Louis, MO |
| | Glass Beads | 0.1 g | 3000E | Potters Industries | Parsippany, NJ |
