# Supplementary figures and images for: Preclinical multimodality phantom design for quality assurance of tumor size measurement
Source: BMC Med Phys. 2011 Sep 30;11:1. doi: 10.1186/1756-6649-11-1 (PMC3206432; doi:10.1186/1756-6649-11-1)

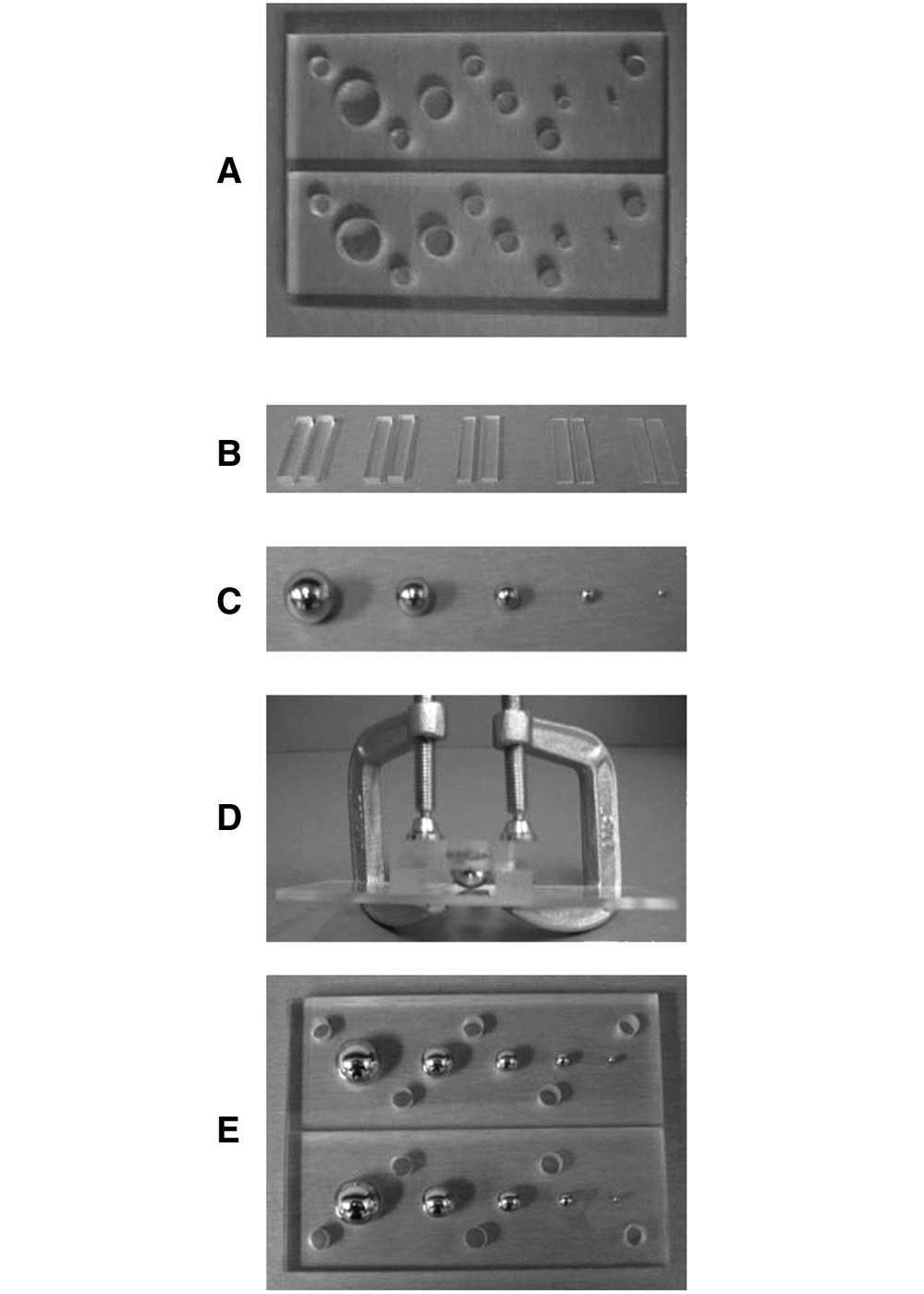

Supplement: Additional file 3 — Lee et al Figure S1.tiff. Preparation for making silicone molds to cast test objects of UTHSCSA multimodality tumor measurement phantom. (A) Two identical base plates, (B) spacer pairs, (C) steel balls, (D) procedure for gluing steel balls, and (E) two identical mirror image base plates with steel balls. [file 1756-6649-11-1-S3.TIFF]

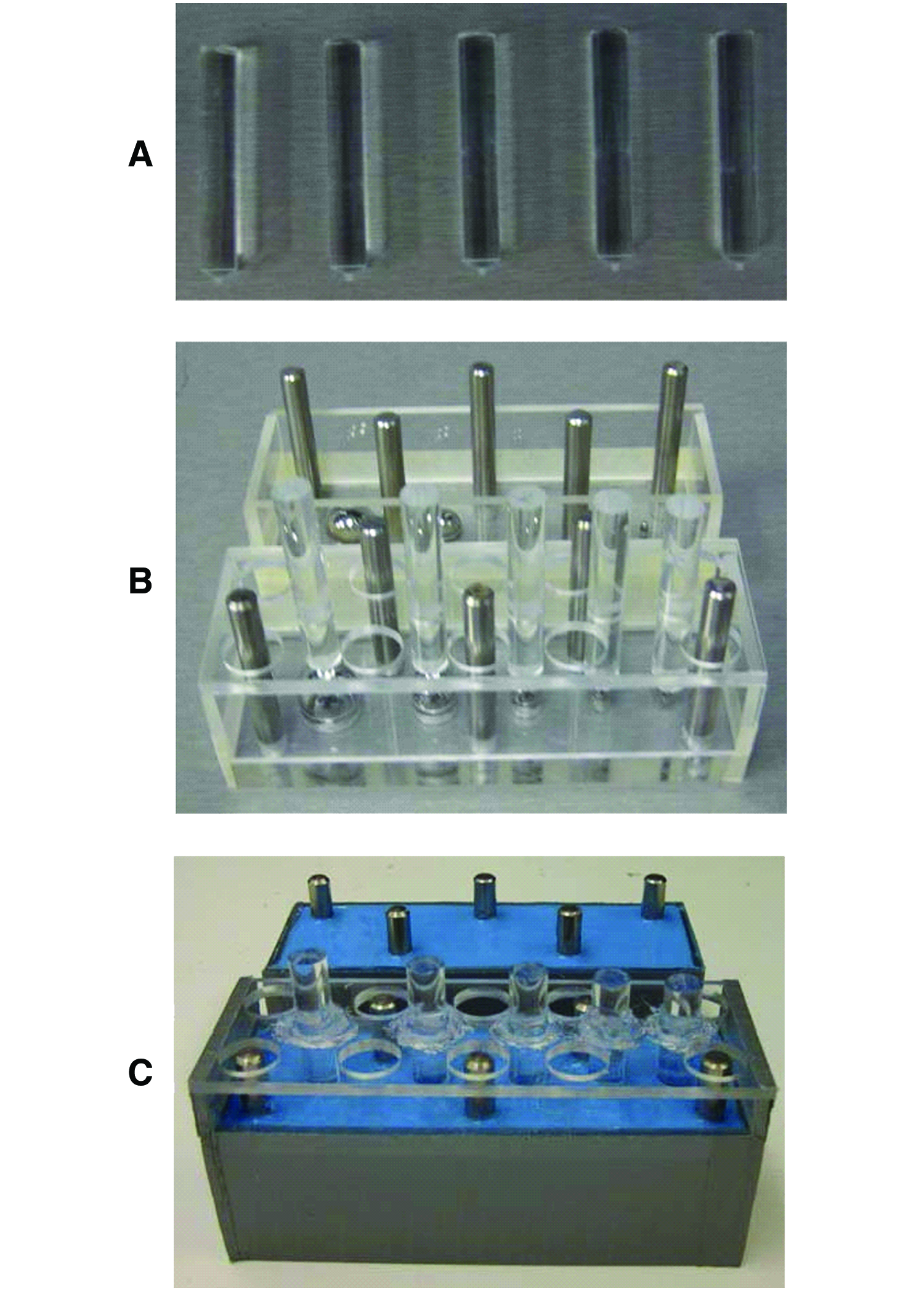

Supplement: Additional file 4 — Lee et al Figure S2.tiff. Materials and procedures for making silicone molds. (A) Acrylic rods with 1 mm tips, (B) base plates with fences, alignment rods, acrylic rods, and (C) mold after addition of silicone rubber compound. [file 1756-6649-11-1-S4.TIFF]

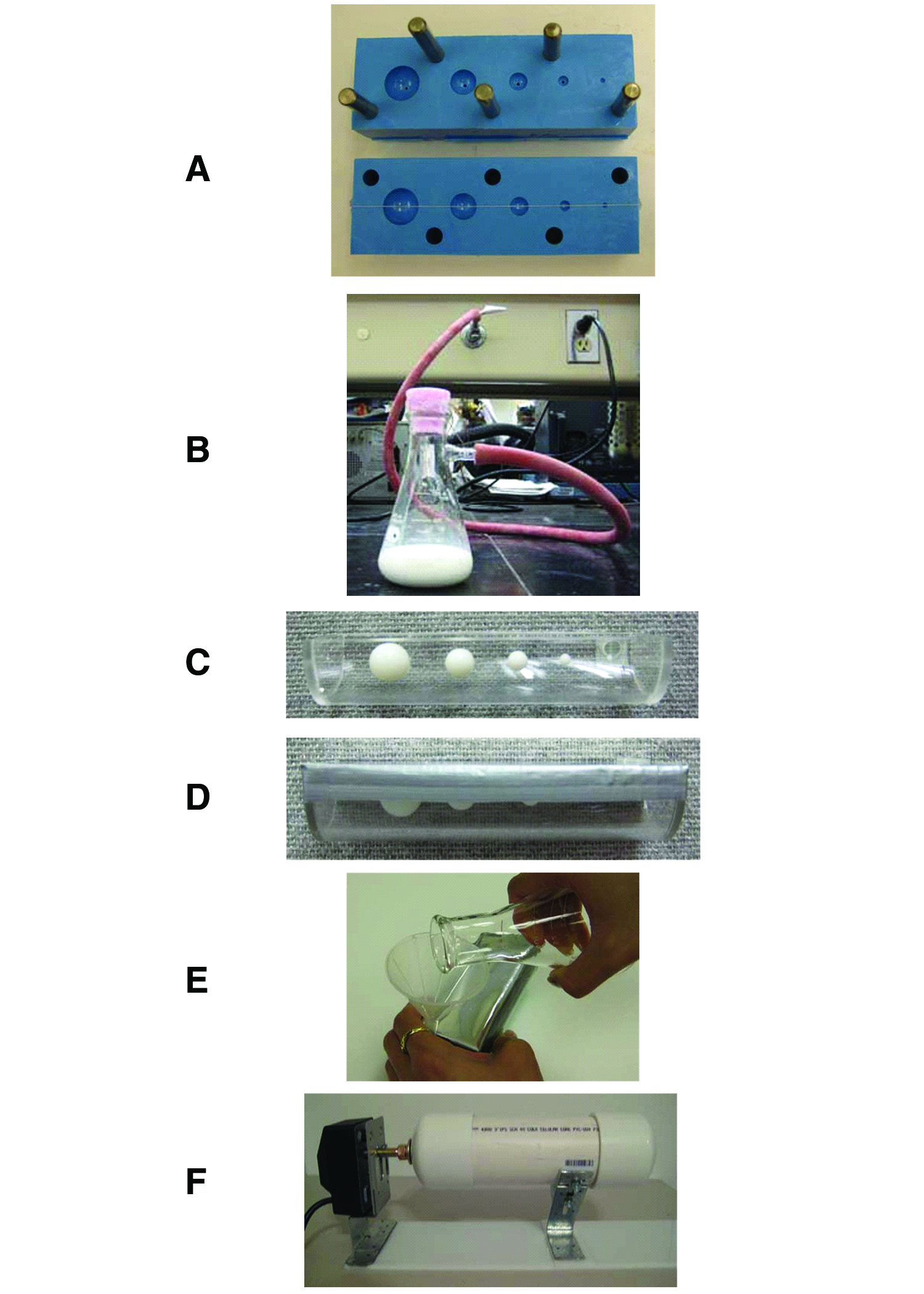

Supplement: Additional file 5 — Lee et al Figure S3.tiff. Procedures for preparing UTHSCSA multimodality tumor measurement phantom using silicone molds. (A) Nylon thread was attached to silicone mold and the molds were adhered with silicone grease. (B) Milk mixture was degassed using house vacuum. (C) After casting test objects in the mold, test objects were mounted in an acrylic container. (D) The top of the container was sealed with surface membrane. (E) Background material was poured into the container. (F) The assembled phantom was placed in rotator and rotated to prevent gravitational sedimentation of tissue mimicking materials. [file 1756-6649-11-1-S5.TIFF]
